# Supplementary material for: Angiotensin 1–7 Stimulates Proliferation of Lung Bronchoalveolar Progenitors—Implications for SARS-CoV-2 Infection
Source: Cells. 2022 Jul 2;11(13):2102. doi: 10.3390/cells11132102 (PMC9266020; doi:10.3390/cells11132102)
Supplement: Supplementary file 1 [file cells-11-02102-s001.zip › cells-1738207-supplementary.pdf]

Supplementary Tabel S1. List of primer sequences used for Real-Time qPCR analysis of murine AT2 cells, BASCs and lung organoids.

**Name of the gene**	**Primer**	**Sequence**
β2M	forward	5’-CATACGCCTGCAGAGTTAAGCA-3’
	reverse	5’-GATCACATGTCTCGATCCCAGTAG-3’
ACE2	forward	5’-TTCTGGGCAAACTCTATGCTG-3’
	reverse	5’-CTCGTGATGGGCTGTCAAG-3’
CD147	forward	5’-GGCAAGTATGTGGTGGGTATCC-3’
	reverse	5’-GATGGTTTCCCGAGTAGTGC-3’
TOP	forward	5’-AAAGATTCCCTGAAGCCTGAA-3’
	reverse	5’-GTTGAAGTCGATGCACAGCA-3’
NEP	forward	5’-TGGGACTACATCAGAAACTGC-3’
	reverse	5’-CGTGCTTGCTCTCTCCAG-3’
AT1	forward	5’-GCTTCCTGTTCCCTTTCCTA-3’
	reverse	5’-TCATTTCTTGGCTTGTTCTTCT-3’
AT2	forward	5’-GATCTGGTGCAGTTACATCTCAG-3’
	reverse	5’-CTTACTCAGCTCCCGCATG-3’
AT3	forward	5’-ACATTCTGGGCTTCGTGTTC-3’
	reverse	5’-TGTCATCATTCCTTGGCGTA-3’
MAS	forward	5’-TTCAGTTGGAAGCGGAGTTT-3’
	reverse	5’-TGGCACAGAGACCTGCTACA-3’
TMPRSS2	forward	5’-GGATTGTGGGTGGATTGAA-3’
	reverse	5’-GCTGCTGAGGGGTTCTTC-3’
KDR	forward	5’-AGGCTTTACTCTCCCCAGTT-3’
	reverse	5’-GGGCTCAGAATCACATCATAA-3’
REN	forward	5’-GCGAGATTGGCATCGGTA-3’
	reverse	5’-TCCCACAAGCAAGGTAGAGG-3’
AGT	forward	5’-AGCATCTCGGTGTCTGTGC-3’
	reverse	5’-AGCAGGGTGGCTCTCTCAC-3’
ACE	forward	5’-GGTAGTGCCTTTCCCAGACA-3’
	reverse	5’-GACTCCGCCCAGAACTCAG-3’
NLRP3	forward	5’-ACCAGCCAGAGTGGAATGAC-3’
	reverse	5’-ATGGAGATGCGGGAGAGATA-3’
CASP1	forward	5’-GCTTTCTGCTCTTCAACACC-3’
	reverse	5’-AAAATGTCCTCCAAGTCACAAG-3’
IL-1 beta	forward	5’-AGTTGACGGACCCCAAAAG-3’
	reverse	5’-CTTCTCCACAGCCACAATGA-3’
IL-18	forward	5’-ACAACTTTGGCCGACTTCAC-3’
	reverse	5’-GTCTGGTCTGGGGTTCACTG-3’
GSDM	forward	5’-CTGGGTCTTGCTAGAAGAATGTGG-3’
	reverse	5’-CTGGCCTAGACTTGACAATAGGAAC-3’
S100A9	forward	5’-TGGTTGGAAGCACAGTTGG-3’

	reverse	5'-CATCAGCATCATACACTCCTCAA-3'
NR1D1	forward	5'-TGGCCTCAGGCTTCCACTATG-3'
	reverse	5'-CCGTTGCTTCTCTCTCTTGGG-3'
AIM2	forward	5'-AGGCAGTGGGAACAAGACAG-3'
	reverse	5'-GAAAACTTCCTGACGCCACC-3'
HMGB1	forward	5'-GGAGGAGCACAAGAAGAAGC-3'
	reverse	5'-GGGGGATGTAGGTTTTCATTT-3'
